# LNMVSNet: A Low-Noise Multi-View Stereo Depth Inference Method for 3D Reconstruction

**DOI:** 10.3390/s24082400

**Published:** 2024-04-09

**Authors:** Weiming Luo, Zongqing Lu, Qingmin Liao

**Affiliations:** Tsinghua Shenzhen International Graduate School, Tsinghua University, Beijing 100084, China; lwm21@mails.tsinghua.edu.cn (W.L.); luzq@sz.tsinghua.edu.cn (Z.L.)

**Keywords:** multi-view stereo, RGB 3D reconstruction, depth estimation

## Abstract

With the widespread adoption of modern RGB cameras, an abundance of RGB images is available everywhere. Therefore, multi-view stereo (MVS) 3D reconstruction has been extensively applied across various fields because of its cost-effectiveness and accessibility, which involves multi-view depth estimation and stereo matching algorithms. However, MVS tasks face noise challenges because of natural multiplicative noise and negative gain in algorithms, which reduce the quality and accuracy of the generated models and depth maps. Traditional MVS methods often struggle with noise, relying on assumptions that do not always hold true under real-world conditions, while deep learning-based MVS approaches tend to suffer from high noise sensitivity. To overcome these challenges, we introduce LNMVSNet, a deep learning network designed to enhance local feature attention and fuse features across different scales, aiming for low-noise, high-precision MVS 3D reconstruction. Through extensive evaluation of multiple benchmark datasets, LNMVSNet has demonstrated its superior performance, showcasing its ability to improve reconstruction accuracy and completeness, especially in the recovery of fine details and clear feature delineation. This advancement brings hope for the widespread application of MVS, ranging from precise industrial part inspection to the creation of immersive virtual environments.

## 1. Introduction

With the widespread adoption of modern RGB cameras throughout society, a vast amount of RGB imagery is easily captured in our daily lives. Compared with professional 3D scanning devices, RGB sensors are more economical and ubiquitously available, thereby democratizing 3D reconstruction technologies and advancing their development and application. Multi-view stereo (MVS) 3D reconstruction presents a promising method for reconstructing indoor and outdoor scenes from multiple viewpoints. Central to 3D reconstruction, multi-view depth estimation and stereo matching algorithms perform the task of feature matching across multiple images given known camera intrinsic and extrinsic parameters. Here, each pixel in the reference image searches along the epipolar line in the target image, transformed by homography, and the best depth is estimated using the cost volume generated by the lowest matching cost, thus recovering the 3D model of the reconstructed scene.

MVS has garnered significant interest in various fields such as industrial applications, architectural reconstruction, entertainment, and augmented and mixed reality. In the industrial sector, autonomous vehicles and robots utilize MVS technology to understand their surroundings [1,2]. Through 3D reconstruction, they can better recognize obstacles, navigate, and plan routes. In the medical field, MVS technology assists in reconstructing three-dimensional models of human organs from medical images taken from multiple angles, proving invaluable for surgical planning and disease diagnosis [3]. Moreover, MVS is utilized for precisely capturing and reconstructing the 3D shapes of complex industrial parts for product design, quality inspection, and reverse engineering [4]. For urban reconstruction, MVS is employed in urban planning and architecture for creating detailed 3D models of buildings and cityscapes, aiding in the planning, design, and visualization of new projects. Additionally, MVS can reconstruct 3D models of ancient buildings and artifacts [5], aiding in preservation, research, and display. This technology can assist in the restoration and conservation of historical sites, offering possibilities for reconstruction even when they are damaged or destroyed. In entertainment and augmented reality, MVS is often used to create high-quality 3D characters and scenes or in AR and VR [6] applications to create realistic 3D environments and objects, offering immersive experiences as well as healthcare applications.

In MVS 3D reconstruction tasks, the process involves capturing scenes from multiple viewpoints to reconstruct their three-dimensional structure. This task often encounters challenges posed by noise interference, stemming from the sensor’s sensitivity to various factors [7], such as changes in ambient lighting, inconsistencies in camera quality, and motion blur. Noise can not only degrade the quality of the 3D model but also lead to erroneous estimations during the reconstruction process. High-resolution, smooth, and low-noise 3D reconstruction results and depth maps are crucial for ensuring the quality of the reconstruction. Noise interferes with the feature extraction and matching process in images, leading to mismatches and inaccurate depth estimations. This interference reduces the geometric accuracy of the 3D model, causing the reconstructed results to deviate from the true shape of the object. In industrial design and manufacturing, the accuracy of 3D models is critical to ensuring the quality and safety of components. Reduced precision may lead to inaccurate component dimensions, affecting assembly and performance and potentially causing product failures or even safety incidents. Similarly, the low precision induced by noise also poses safety risks in medical applications and autonomous driving, where reduced reconstruction quality can lead to misjudgments.

To compensate for the impact of outliers and noise, algorithms require additional steps to identify and filter out mismatches caused by noise, or to perform post-processing such as smoothing, denoising, and hole-filling. This not only increases computation time but may also require more computational resources.

Many existing methods, while suppressing noise, are still limited in recovering fine-scale details and sharp features. In traditional MVS 3D reconstruction methods [8,9], the approaches often rely on assumptions based on geometric and photometric constraints, such as scene geometric continuity and photometric consistency. In the real world [10,11], these assumptions may not always hold, especially in scenes with complex geometric structures or varying lighting conditions. Traditional MVS methods typically compute cost volumes to represent the confidence level under different depth hypotheses. These cost volumes are usually constructed by comparing image regions from different viewpoints, relying on pixel similarities or consistencies. However, significant errors in cost calculation can arise if there are drastic changes in the frequency domain signals in images, such as changes in lighting, variations in material reflections, or sensor thermal noise.

For learning-based MVS reconstruction methods, the information aggregated using 3D CNNs is theoretically highly sensitive to noise in the input data and inaccuracies in feature matching. Particularly in areas where feature matching is challenging (e.g., low-texture or repetitive texture regions), errors may be amplified because of gain calculations. Pixel-wise MVS 3D reconstruction methods tend to model noise as outliers. To address these limitations, this paper proposes a deep learning network called LNMVSNet, which is designed to enhance local feature attention and enable the fusion of features at different scales for low-noise MVS 3D reconstruction.

In summary, our contributions are as follows:

1. We propose LNMVSNet, a network with low sensitivity to noise, through the introduction of a multi-level depth feature fusion mechanism and a novel attention filtering mechanism. These innovations effectively utilize the varying sensitivities of multi-level features to noise and pixel weight scoring, resulting in noise being less sensitive in the preliminary step of depth estimation in MVS reconstruction.
2. Our LNMVSNet achieved exceptional results on multiple benchmark datasets, yielding smooth and low-noise depth estimates as well as reconstructed point clouds. Additionally, we analyzed the impact of noise on reconstruction evaluation metrics through qualitative experimental results.

## 2. Literature Review

### 2.1. Traditional Multi-View Stereo Methods

In contemporary scholarly discourse, multi-view stereo (MVS) methodologies are stratified based on their modality of scene representation, encompassing volumetric, point cloud, mesh, and depth map strategies.

Volumetric Methods: These methodologies [12,13] instantiate the reconstruction paradigm by partitioning tridimensional space into a meticulously aligned grid of voxels, each voxel being ascribed to a scalar magnitude. This magnitude quantitatively represents the probabilistic occupancy or confidence level of the voxel within the contextual scene. Conceptualized mathematically, volumetric reconstruction is akin to delineating a scalar field $V:R^{3}\to \left[0,1\right]$, wherein the scalar value at each spatial coordinate conjectures the likelihood of the scene’s surface intersecting at that juncture. Predominantly, these methods integrate voxel fusion, spatial hashing, or octree structures to efficaciously manage spatial data. They excel in reconstructing complex and irregular surfaces, albeit at a heightened computational and storage cost.

Point Cloud Methods: Characterized by [14,15], these approaches derive a rudimentary tridimensional structure of a scene via the extraction and juxtaposition of feature points across multiple image vantages, subsequently transposing these points into a conglomerate of spatial coordinates. A point cloud, thus, is denoted as $P=\{p_{i}\in R^{3}\}$, with each point $p_{i}$ encapsulating tridimensional coordinates and potentially ancillary attributes like chromaticity or intensity. The primary objective is the precise restitution of sparse or semi-dense geometric attributes of the scene, though these methods might grapple with the continuity and integrity of surface structures.

Mesh Methods: Extensively discussed in [16,17,18], these techniques not only render the tridimensional points but also articulate the topological interconnects among these points, culminating in polygonal meshes. Formally, a mesh is represented as M=(V, E, F), with V symbolizing the vertex set, E the edge consortium, and F the facet aggregation. These polygons, predominantly triangular, are constituted by vertices, aiming to fabricate continuous and sleek surface models, thereby catering to applications necessitating superior surface reconstruction fidelity. Encompassing surface reconstruction, mesh optimization, and refinement, these methods endeavor to augment the accuracy and aesthetic appeal of the model.

Depth Map Methods: Elaborated in [8,9,19], these techniques revolve around the estimation of per-pixel depth information from multifaceted viewpoints. Each depth map correlates with a specific vantage point, depicting the distance from that point to various loci on the scene’s surface. Expressed mathematically, a depth map can be articulated as a function $\left(D:\Omega \subset R^{2}\to R\right)$, with D(u, v) signifying the depth of the scene point corresponding to the image plane coordinates (u, v). Emphasizing pixel-wise depth continuity, these methods typically employ cost aggregation and global optimization to curtail disparity errors and reconstruction noise. For instance, COLMAP [8] integrates the estimation of pixel-centric view selection, depth maps, and surface normals, harnessing photometric and geometric precepts. Depth-based approaches demonstrate enhanced adaptability in sculpting the tridimensional geometry of scenes. ACMM [9] introduces innovations like multi-scale geometric consistency, adaptive checkerboard sampling, and a multi-hypothesis joint view selection mechanism. Given their structural consonance with the original 2D image data, depth map methods exhibit computational efficiency, particularly for extensive scenes and high-resolution imagery. Furthermore, the resultant output seamlessly integrates with extant 2D image processing paradigms, facilitating post-processing activities like depth map fusion, filtering, and optimization. Hence, this paper, in consideration of the deployability and efficacy of MVS methods in practical applications, adopts a Learning-Based Depth MVS baseline as its foundational strategy.

### 2.2. Learning-Based Multi-View Stereo Method

While traditional MVS methods have yielded impressive outcomes, their reliance on manually engineered features renders them suboptimal for non-Lambertian surfaces. The conventional paradigm’s assumption of photometric consistency is particularly unreliable in areas with low or no texture. Recent strides in MVS research have moved beyond traditional handcrafted image features, embracing deep learning (DL) to achieve enhanced reconstruction precision and completeness. Like their traditional counterparts, DL-based methods can also be categorized based on different scene representation techniques.

In volumetric methods, solutions like SurfaceNet [20] and LSM [21] construct a cost volume using multi-view images and employ 3D CNNs for regularization and voxel inference. However, because of the inherent limitations of volumetric representations, SurfaceNet and LSM are confined to small-scale reconstructions, with limited computational capabilities for larger scenes. In contrast to SurfaceNet and LSM, depth-based MVSNet [22] has improved MVS reconstruction performance through depth map estimation. MVSNet, processing a reference image along with multiple source images, extracts depth image features and encodes camera geometry within the network through a differentiable unit, constructing a three-dimensional cost volume. To mitigate the substantial memory consumption of MVSNet, several variants have been proposed and categorized into multi-stage and recursive methods. CasMVSNet [23], CVP-MVSNet [24], EPP-MVSNet [25], and PatchmatchNet [26] adopt a coarse-to-fine strategy, initially predicting low-resolution depth maps with large depth intervals and iteratively upsampling and refining depth maps with narrower depth ranges. Although the coarse-to-fine architecture successfully reduces memory usage, it is not conducive to high-resolution depth reconstruction because of potential inaccuracies in coarse-level depth predictions. Consequently, recursive methods such as R-MVSNet [27] and D2HC-RMVSNet [28] have been proposed. They sequentially regularize cost maps along the depth dimension with a cyclical network, inferring depth maps across a vast depth range. Recognizing the smooth nature of cost volume regularization by 3D CNNs, [29] introduced an Edge-Preserving Multi-view Stereo Network (EPNet) for practical depth estimation, reinforcing the edges in depth estimation.

Previous works have made significant contributions in terms of high-resolution and efficient utilization of computational power. With the recent advancements in deep learning for 2D and 3D depth estimation from RGB sensor data [30,31,32,33], depth map methods have demonstrated more robust performance compared with other MVS reconstruction approaches, particularly in handling complex scenes and challenging lighting conditions. NTPP-MVSNet [34] explored the specific role of depth sampling in MVS reconstruction networks by utilizing the normal and depth information of adjacent pixels to propagate tangent planes, highlighting the significant role of depth information in the 3D reconstruction process. However, depth map methods, calculating depth for each pixel, are generally more sensitive in capturing scene details, especially surface textures and edges, compared with point cloud or volumetric methods. Previous works focused on the precision of per-pixel level operations yet overlooked the converse aspect: undesired, random, or systematic errors introduced during the acquisition and processing stages are also modeled by depth estimation networks. EPP-MVSNet also pointed out that real-world MVS reconstruction is challenging because of noise. These errors may originate from various factors such as inherent noise in image sensors, changes in environmental lighting, reflective properties, limitations of imaging equipment, and inaccuracies in feature extraction and matching algorithms. Noise ultimately manifests as random fluctuations in image data or discordant points in 3D reconstruction results, affecting the accuracy of depth estimation and the quality of the final 3D model. Some works have implemented modest measures to reduce noise, such as MVSNet, which further suppresses reconstruction noise by determining the visible views for each pixel in the depth map to 3D point cloud conversion process and averaging all reprojected depths, and PatchmatchNet, which has attempted to incorporate anti-noise training strategies to combat the impact of noise; however, these actions that do not qualitatively analyze and eliminate noise do not fundamentally alter the sensitivity of depth map MVS three-dimensional reconstruction to noise. A low-noise MVS depth estimation and reconstruction network is urgently needed to address the high precision requirements of industrial production.

## 3. Motivation and Contribution

To address the limitations of previous works, it is imperative to precisely define noise and identify its underlying causes. The models generated during 3D reconstruction typically manifest several characteristic noise features. Initially, outliers, a prevalent phenomenon, manifest as isolated points distinctly separated from the main structure, either appearing singly or forming scattered clusters. Moreover, the reconstructed surfaces may exhibit surface roughness, leading to uneven elevations in areas that should be smooth. Furthermore, the reconstruction models may suffer from the loss of surface detail, wherein subtle surface features fail to be accurately reconstructed. Ghost structures, another common occurrence, are structures that do not exist in the original scene but appear in the reconstruction model because of occlusion or mismatching. Holes, generally forming in areas with poor observational conditions or missing data, reflect information loss during the reconstruction process. Lastly, the issue of inconsistent density in point clouds is manifested by a significant disparity in the distribution density of points across different areas. The presence of these noise features significantly constrains the quality and accuracy of the reconstructed models.

Noise sources in 3D point clouds encompass sensor noise stemming from inherent defects in imaging sensors to noise in depth maps predicted through multi-view depth estimation. In the source image segment, unavoidable quantization steps in the digital imaging process introduce quantization errors, and geometric distortions caused by lens optical characteristics also pose challenges to the reconstruction process. Variations in illumination and shadow effects can cause significant visual discrepancies among different images, thus disrupting feature matching. The reflective and transmissive errors generated by objects with complex reflective properties during imaging, as well as color distortions resulting from inaccurate camera color calibration or changes in environmental light sources, impact the accuracy of feature extraction.

**Figure 1:**
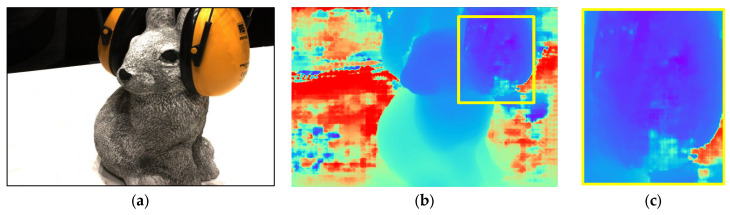
Visualization example of noise in multi-view stereo depth estimation: (**a**) Source image; (**b**) Depth map with noise; (**c**) Noise visualization.

In MVS 3D reconstruction methods based on depth maps, the noise directly affects the quality of the final 3D point cloud, as the reconstruction of point clouds entirely utilizes the depth map to supplement the three-dimensional coordinates for each pixel of the 2D image. The noise in depth maps, which is shown in Figure 1, as an example, primarily manifesting as inaccurate depth values, leads to incorrect spatial positioning when converted to 3D point clouds, thereby generating noise points. The noise in source images, blurriness, or low contrast can affect the accuracy of depth estimation, leading to deviations between the actual points in the image and their predicted projection positions in three-dimensional space. Image quality issues like noise, blurriness, or low contrast in the source images directly impact the accuracy of depth estimation. Accurately estimating disparity in areas lacking texture or with complex regions is exceptionally challenging, often leading to noise in depth estimation. Different lighting conditions and surface reflective properties can also cause appearance variations in the same object under different viewpoints, increasing the errors in depth estimation.

Thus, inspired by the deficiencies and strengths in prior research, we pose the following research question: “How can we effectively reduce the noise in MVS depth estimation and enhance the accuracy and quality of 3D reconstruction models?” In our paper, we introduce a low-noise MVS depth estimation and reconstruction network named LNMVSNet and employ the following three solutions to achieve low-noise depth maps and point clouds, obtaining outstanding results on the DTU [10] and Tanks and Temples [11] benchmark datasets. The core solutions are as follows:

1. We incorporated a mechanism for the fusion of depth map features at different scales, effectively diminishing the influence of noise on the final reconstruction results. Features at varying scales have their respective advantages in handling noise; by integrating these features, we achieve complementarity and reduce error propagation, making the overall reconstruction process more robust.
2. During the cost volume regularization process, we utilized an attention-based filter with a noise-aware mechanism for selecting and emphasizing important features while suppressing irrelevant or noisy components. Through this weighted allocation, the network can focus more on significant signals, thereby reducing the impact of noise.

## 4. Method

**Figure 2:**
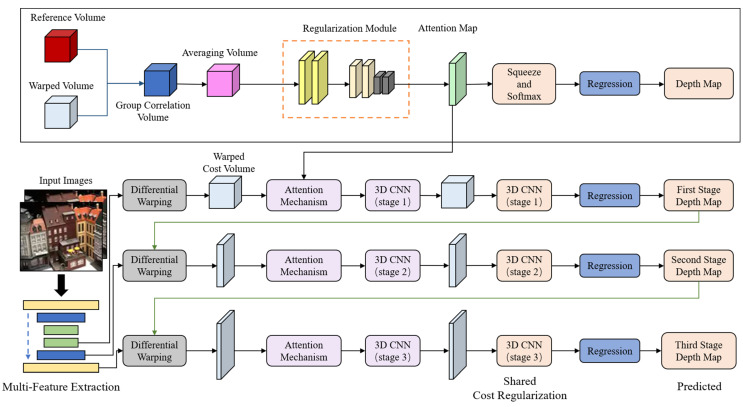
General pipeline of the proposed LNMVSNet structure.

This section describes the detailed architecture of the proposed LNMVSNet. Herein, we employ the representative MVSNet [22] and CasMVSNet [23] as backbone networks and adopt a cascaded cost volume for multi-view stereo and stereo matching. Figure 2 illustrates the architecture of LNMVSNet. For the task of MVS reconstruction, the core objective is to obtain a high-quality depth map. Our depth estimation network is divided into the following five parts: multi-view image feature extraction, cost volume construction, cost volume regularization, probabilistic cost volume, and depth regression. We emphasize the construction of the cost volume and multi-level feature fusion to refine the entire MVS depth estimation process and achieve low-noise reconstruction.

### 4.1. Cascaded Structure

The backbone network section adopts the cascaded structure proposed by CAS-MVSNet. The method CAS-MVSNet employs for implementing cascading operations involves constructing a multi-stage network architecture, refining depth (or disparity) estimation at each stage. Initially, a coarse depth map is estimated using a smaller cost volume, which allows for a reduction in the hypothesis space for depth at the current resolution based on the depth map output from the preceding level. Our LNMVSNet employs a three-level cost volume hierarchy for depth map estimation, which includes two intermediate results and one final depth output. The working mechanism is detailed as follows: At stage k, the network defines a depth (or disparity) hypothesis range $R_{k}$. This range is computed based on the output of the previous stage, which is:

(1) $$R_{k+1}=R_{k}\cdot w_{k}$$

where $w_{k}<1$ and represents a factor reducing the hypothesis range.

Compared with traditional single-level cost volumes, the initial hypothesis plane interval $I_{k}$ is set larger, generating a coarse depth (or disparity) estimation. In subsequent stages, finer output is produced by refining the hypothesis plane interval:

(2) $$I_{k+1}=I_{k}\cdot p_{k}$$

where $p_{k}<1$ and is reducing factor of hypothesis plane interval.

Then, at stage k, the number of hypothesis planes is determined by dividing the hypothesis range $R_{k}$ by the hypothesis plane interval:

(3) $$D_{k}=R_{k}/I_{k}$$

The spatial resolution at each stage is doubled from the previous one, achieved by doubling the resolution of input feature maps. Therefore, the total resolution is defined as

(4) $$\frac{W\times H}{2^{N-k}}$$

where N is 3 in multi-view stereo tasks and 2 in stereo matching tasks.

A warping operation applies the cascaded cost volume computation to map the disparity learned at stage k+1, formulated as:

(5) $$H_{i}\left(d_{m}^{k+1}\right)={K^{\prime }}_{i}\cdot {R^{\prime }}_{i}\cdot \left(\frac{\left(1-t_{i}\right)\cdot m^{\prime }}{d_{m}^{k}+\Delta m_{k+1}}\right)\cdot R_{k}^{T}\cdot K_{i}^{-1}$$

where $d_{m}^{k}$ represents the predicted depth of the $m^{th}$ pixel at stage k, ${K^{\prime }}_{i}$ means the transformed version of intrinsic matrix K, and ${R^{\prime }}_{i}$ represents the updated version of rotation matrix R of $i^{th}$ step. $t_{i}$ is a transformation parameter, related to translation applied to the image features.

Through these cascading steps, our backbone structure progressively narrows the search range and hypothesis plane interval at each stage, ultimately producing a precise depth map. This approach effectively reduces computational load while maintaining fine estimation of high-resolution depth maps. 

### 4.2. Depth Feature Sharing

**Figure 3:**
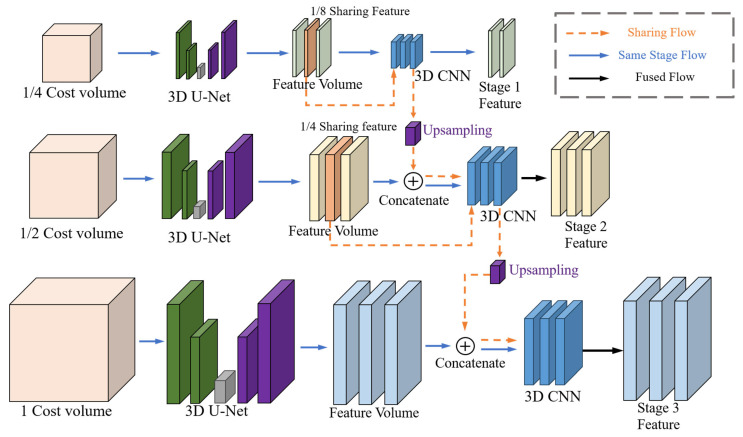
Detail structure of depth feature sharing.

Within the cascaded structure, as each level increases the spatial resolution, this implies that any noise or errors present at the initial stages will be amplified in subsequent levels. Moreover, each level relies on the output of the previous stage, leading to the propagation and amplification of noise from initial estimations through the levels. To compensate, the hypothesis depth range at each cascaded stage is reduced. This approach enhances the accuracy of depth estimation but also implies that if the initial stage’s estimation is inaccurate, subsequent stages will lack the capability to correct these errors, as they search for the correct depth within a smaller range. To mitigate the high sensitivity to noise of the cascaded backbone structure, LNMVSNet introduces a feature fusion mechanism, as displayed in Figure 3. In the backbone cost volume regularization part, the input is H×W×C×D, where D is the number of depth values sampled (we use sampling numbers of 48, 32, and 8). The cost volume, after being regularized through a 3D U-Net [35] structure, involves intermediate processing to obtain 1/8 and 1/4 scale feature volumes, which are then connected via upsampling to the next stage, with additional 3D convolution layers reducing the feature channels to a fixed size. Notably, the D dimension of Feature volumes across the three stages varies, as does the number of channels. Therefore, the H×W×C×D features intended for concatenation are first processed through a 3D convolution layer to align the D dimension with $D_{1}$ of the next stage before concatenation.

In addressing noise within high-resolution feature processing, there exists a propensity for such features to misinterpret noise as substantive detail, leading to the model’s erroneous amplification of noise. In contrast, features of lower resolution, by virtue of their encompassing global attributes, are capable of providing a stream of information that is inherently smoother. This characteristic is instrumental in enabling the model to discount noise. Consequently, a depth feature concatenation strategy is applied to amalgamate information across disparate dimensions, thereby preserving detail fidelity while simultaneously mitigating the unwarranted magnification of noise. The pre-concatenation processing of features from distinct stages via a 3D convolutional layer—ensuring the congruence of depth (D dimension) and channel count (C dimension) with the ensuing stage—is predicated on the 3D convolutional layer’s inherent capacity for spatial feature extraction and data smoothing. This capability is pivotal in attenuating or eliminating noise. Such meticulous adjustment confers the following dual advantages: firstly, it endows the subsequent level with a representation of features that is markedly precise; secondly, by expurgating noise, it forestalls the compounding and escalation of erroneous signal interpretations.

The mathematical expression for the feature fusion part is as follows:

Let $F_{k}$ represent the feature volume at stage k. In order to match the depth dimension, we utilize a 3D convolutional layer to adjust the depth dimension $D_{k}$ of stage k to $D_{k+1}$.

(6) $$F_{k}^{\prime }=3\mathrm{DConv}(F_{k},D_{k+1})$$

where *3DConv*(⋅) signifies the 3D convolutional processing with the target depth $D_{k+1}$. For stage k+1, the scale adjustment and feature fusion can be expressed as:

(7) $$F_{k+1\_new}=\mathrm{Concat}(F_{k+1},\;\mathrm{Upsample}\left(F_{k}^{\prime }\right))$$

where $F_{k}^{\prime }$ is the feature volume processed by a 3D convolutional layer and $F_{k+1\_new}$ is the feature volume at stage k+1 concatenated with a feature from stage k. Upsample (⋅) denotes the up-sampling operation and $\mathrm{Concat}\left(\cdot \right)$d represents the concatenation operation. 

Finally, the adjusted feature volume is concatenated with the feature volume of the next stage, followed by an additional 3D convolutional layer to reduce the number of feature channels to a fixed size:

(8) $$F_{k+1\_new}^{\prime }=3\mathrm{DConv}(F_{k+1\_new})$$

These steps articulate how the model’s performance is enhanced through the feature fusion component on top of the original cascaded structure. This cascading and fusion approach allows for maintaining resolution while reducing computational complexity and increasing the accuracy of depth estimation.

### 4.3. Cost Attention Mechanism

**Figure 4:**
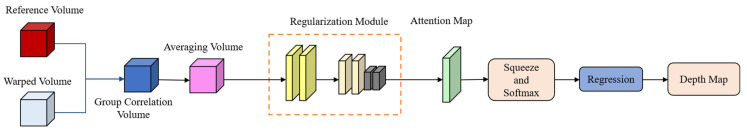
Detail structure of attention mechanism.

LNMVSNet incorporates a unique attention mechanism after the differential deformation module to ensure that the features passed to the 3D CNN are most beneficial for the final task. Features “noticed” through this attention mechanism are then fed into the 3D CNN for in-depth processing. The specific module details are as shown in Figure 4.

Perspective Mapping: The first step involves mapping from the source perspective to the reference perspective. The purpose of this step is to align image information from different viewpoints to facilitate the subsequent steps of effective comparison and merging of this information. By mapping the image from the source perspective to the reference perspective, a unified reference framework is created, allowing information from different viewpoints to be compared and processed in the same spatial context. After the mapping, the model generates the warped volume and the reference volume, representing the image features of the source and reference perspectives, respectively. This step prepares for the subsequent construction of the cost volume by providing image features for each perspective.

Group Correlation Processing: Full Correlation (FC) has been widely used to build the cost volume. In the Cost Attention Mechanism, this is replaced with group correlation (GC) processing. This approach aims to compare and merge image features from different perspectives more effectively by measuring the differences between perspectives through correlation, aiding in subsequent depth estimation. GWCNet [36] considers FC to be an effective method for measuring feature similarity, but it loses a lot of information as it generates a single channel correlation map for each disparity level. In R-MVSNet [27], the GC operation is also proven effective.

Specifically, GC works by calculating the correlation between a set of image features, which can effectively avoid calibration error. In multi-view image processing, each viewpoint provides different information about the scene. GC assesses the correlation between image features from these different perspectives, determining which features are similar and which are different. This comparison is achieved by calculating the correlation coefficient between features, with a high coefficient indicating high similarity, and vice versa. The GC operation process is threefold: First, features are extracted from the image of each perspective. These features can include the image’s color, texture, edges, etc. Secondly, these features are grouped, each group containing features from different perspectives. The features in these groups are then compared. Eventually, for each pair of features, their correlation weight is calculated.

The fundamental mathematical idea of GC is to divide features into several groups and calculate the correlation mapping for each group. The division of features into groups, the calculation of correlation, and how to organize the correlation mappings into the shape and size of a matching cost volume will be derived in the following sections. Specifically, the channel count of unary features is denoted as $N_{c}$. All channels are evenly divided into $N_{g}$ groups, and along the channel dimension, each feature group thus has $\frac{N_{c}}{N_{g}}$ channels. The g−th feature group $f_{l}^{g}$, $f_{r}^{g}$ contains the original features $f_{g}$, $f_{r}$ of the channel group of $\left[(\frac{gN_{c}}{N_{g}},\frac{gN_{c}}{N_{g}}+1,...,\frac{gN_{c}}{N_{g}}+(\frac{N_{c}}{N_{g}}-1)\right]$. The formula for calculating group correlation is as follows:

(9) $$C_{gwc}(d,x,y,g)=\frac{1}{N_{c}/N_{g}}\langle f_{l}^{g}(x,y),f_{r}^{g}(x-d,y)\rangle$$

In Equation (9), ⟨⋅,⋅⟩ denotes the inner product. Note that correlation is calculated for all feature groups g as well as all disparity levels d. Then, all correlation mappings are packed into a matching cost volume, shaped as $\left(D_{max}/4,H/4,W/4,N_{g}\right)$, where $D_{max}$ represents the maximum disparity and $\left(D_{max}/4\right)$ corresponds to the feature’s maximum disparity. When $N_{g}=1$, group correlation returns back to full correlation.

Average Operation: Following the GC process, we employ an averaging approach to process the cost volume instead of the commonly used variance mechanism. This modification aids in reducing noise and errors, thereby making depth estimation more precise. Averaging is typically simpler and more efficient computationally compared with variance calculation. It also offers more stability when handling feature data from different viewpoints. Since variance is a measure of data spread, it is highly sensitive to outliers or noise. If an image from a certain viewpoint is affected by noise, lighting changes, or occlusions, these outliers may be amplified in variance calculations, thus affecting the accuracy of the final depth estimation. Moreover, variance is significantly influenced by data distribution; large differences in image features between viewpoints can result in high variance values, leading to instability in constructing the branch cost volume. An unstable cost volume increases the uncertainty in depth estimation, reducing accuracy. In terms of computational cost, variance calculation is relatively complex, requiring the computation of each data point’s deviation from the mean, followed by the averaging of these squared deviations. In processing large datasets, this efficiency improvement can significantly accelerate the overall depth estimation process.

Branch Cost Volume Regularization: The cost volume in the attention mechanism branch is processed using 3D CNNs, aiming to further enhance the accuracy of depth estimation. Three-dimensional CNNs can integrate surrounding spatial information, aiding the model in identifying and inferring continuous surfaces and object boundaries, thereby producing smoother and more accurate results in the depth map. Our regularization process involves applying multiple 3D convolutional layers on the cost volume. Each layer seeks to learn and extract spatial contextual information, which helps differentiate foreground from background, eliminate noise, and address issues like occlusions and texture repetition. Through regularization, uncertainties and noise in the estimation are reduced, yielding a smoother and more accurate depth map.

Attention Map: Following the preceding steps, the system predicts the depth value for each pixel, along with a corresponding attention weight map. After processing through the 3D CNN, the network outputs an attention weight map. This map essentially represents the weights in the convolutional calculations, with each value corresponding to a pixel in the input image. Each weight in the weight map signifies the importance of that pixel in depth estimation. Regions with higher weights indicate their greater importance in depth estimation and should be given more attention. Conversely, areas with lower weights in LNMVSNet are, as previously defined, considered noise.

In processing the cost volume, the weight map adjusts the importance of each pixel, essentially applying weights. The weighted cost volume is then fed into a filter, which uses the information from the weight map to determine how to process different data points, preserving more details in high-weight areas and smoothing low-weight areas to reduce noise. A key feature of the attention mechanism is its dynamic adjustment based on the input data. Thus, throughout the reconstruction process, the weight map can be real-time adjusted according to different input scenes or data characteristics, enabling the filter to process data more intelligently and flexibly.

The mathematical principle behind the attention mechanism lies in calculating the attention scores $a_{i}$, where i represents the index of the feature vector. Firstly, we need to normalize these scores to ensure their sum equals 1, accomplished by using the *softmax* function:

(10) $$a_{i}^{\prime }=\frac{exp\left(a_{i}\right)}{\sum_{j}exp\left(a_{j}\right)}$$

The normalized attention scores $a_{i}^{\prime }$ are then used to weight the feature vectors $f_{i\;}$, resulting in the weighted feature vectors $f_{i}^{\prime }$:

(11) $$f_{i}^{\prime }=a_{i}^{\prime }\cdot f_{i\;}$$

Since the cost volume is an abstract representation obtained from 3D CNN computations, it is not possible to visualize the depth map directly. However, we will demonstrate the effectiveness of the attention mechanism in our Experiment Section by showing the noise metrics of our reconstructed depth maps. It is important to note that the cost volume framework in the attention module is not the same concept as the cost volume in the main backbone. This branch cost volume is transformed through the attention mechanism module to act as a weight and supervise the main backbone cost volume. Similarly, the depth map prediction within the attention mechanism serves only to form a supervisory pattern in the sub-branch and does not have a direct connection with the main backbone’s depth map.

### 4.4. Depth Map Filtering and Fusion

Upon obtaining the depth maps, these maps are leveraged for three-dimensional reconstruction. Initially, to transmute the results into a dense point cloud, it is imperative to sift through and eliminate anomalies found within the background and occluded regions. In the aspect of depth map fusion, akin to various multi-view stereo methodologies, a procedural step for depth map fusion is adopted to amalgamate depth maps from disparate viewpoints into a cohesive point cloud representation. LNMVSNet further enhances this process by integrating a step for edge-preserving filtration. Following the fusion of depth maps, the use of edge-preserving filters, notably the Bilateral Filter, facilitates the diminution in noise while concurrently preserving the acuity of image edges, thereby augmenting the quality of the resultant 3D point cloud. The Bilateral Filter, a quintessential non-linear filtering technique, has been substantiated to exhibit commendable efficacy in depth map processing [37] and feature fusion contexts [38]. It contemplates the spatial proximity and the disparity in pixel values among pixels, efficaciously smoothing the image whilst retaining edge integrity. The operation of the Bilateral Filter is mathematically articulated as follows:

(12) $$D_{p}^{\prime }=\frac{1}{W_{p}}\sum_{q\in S}G_{\sigma_{s}}\left(∥p-q∥\right)G_{\sigma_{r}}\left(\left|D_{p}-D_{q}\right|\right)D_{q}$$

In the bilateral filtering formulation (12) for depth map estimation, the following elements are included: $D_{p}^{\prime }$ represents the filtered depth value at location p; $D_{p}$ and $D_{q}$ are the original depth values at locations p and q, respectively; s denotes the neighborhood centered around p; $G_{\sigma_{s}}$ is a spatial Gaussian function, employed to gauge the spatial proximity between locations p and q, with $\sigma_{s}$ being the standard deviation of the spatial kernel; $G_{\sigma_{r}}$ is a range Gaussian function, used to assess the similarity between depth values $D_{p}$ and $D_{q}$, where $\sigma_{r}$ is the standard deviation of the range kernel; and $W_{p}$ is a normalization factor, ensuring that the brightness level of the filtered depth map remains consistent. 

This formulation allows the Bilateral Filter to reduce noise in the depth map while maintaining the clarity of object edges, thereby providing more accurate depth information for subsequent 3D reconstruction.

The culmination of this process involves taking the aggregate of all reprojected depths as the definitive depth estimation for each pixel. Subsequently, the amalgamated low-noise depth maps are directly reprojected into the spatial domain to fabricate a 3D point cloud with low noise.

## 5. Experiment

### 5.1. Dataset Description

We conducted our experiment on benchmark datasets similar to other methods. The DTU Dataset [10] comprises an extensive collection of MVS data, encompassing 124 different scenes captured from either 49 or 64 perspectives across seven distinct lighting environments. DTU offers 3D point clouds generated through structured light sensor technology. Every perspective is accompanied by a corresponding image and precisely calibrated camera parameters. Conversely, the Tanks and Temples dataset [11] features a variety of scenes, both indoor and outdoor, set under authentic lighting conditions and exhibiting significant scale diversity. To benchmark against alternative methodologies, LNMVSNet conducts evaluations of its outcomes on the intermediate subset of this dataset. In our evaluation, we employed standard distance metrics, namely, accuracy (Acc.) and completeness (Comp.), to assess the quality of reconstructed point clouds on the DTU dataset. For the Tanks and Temples dataset, however, we utilized percentage-based measures of accuracy and completeness. The dataset score was determined by calculating the mean of the average accuracy and the average completeness.

### 5.2. Quantitative DTU Results

To explore the results, we selected mainstream traditional methods and state-of-the-art (SoTA) deep learning approaches for a comparative evaluation against our LNMVSNet. The performance of different multi-view stereo (MVS) 3D reconstruction networks on the DTU dataset is showcased in Table 1. Initially, focusing on conventional MVS techniques, such as Tola [15] and Furu [10] depicted in the table, their performance in terms of accuracy and completeness is typically suboptimal. Despite achieving relatively favorable overall scores of 0.766 mm and 0.775 mm, respectively, the efficacy of these methods is constrained in complex scenes, often because of their lack of capability in effectively handling highly nonlinear and intricate data distributions. While stable, these traditional approaches exhibit limitations in capturing fine details and reconstructing complete structures, which is reflected in the precision and completeness of the 3D models.

It is distinctly evident that deep learning methods have played a pivotal role in enhancing the quality of 3D reconstruction. When comparing traditional techniques with those based on deep learning, the performance of LNMVSNet across various metrics is particularly noteworthy. A notable leap in performance is observed as we pivot to deep learning-based approaches. LNMVSNet obtains an accuracy metric of 0.305 mm, implying that its generated 3D models maintain low noise in complex scene reconstructions. More crucially, LNMVSNet surpasses all other methods with an overall score of 0.308 mm, representing the optimal balance between accuracy and completeness, ensuring that the model captures intricate details precisely while also preserving the integrity of the whole structure. While MVSNet and CasMVSNet serve as the foundational backbone networks in our study, LNMVSNet significantly surpasses their performance because of its high-efficiency denoising effects. The enhanced capability to filter out noise and artifacts in the data contributes to the superior accuracy and completeness metrics demonstrated by LNMVSNet, allowing it to outperform these established methods.

**Figure 5:**
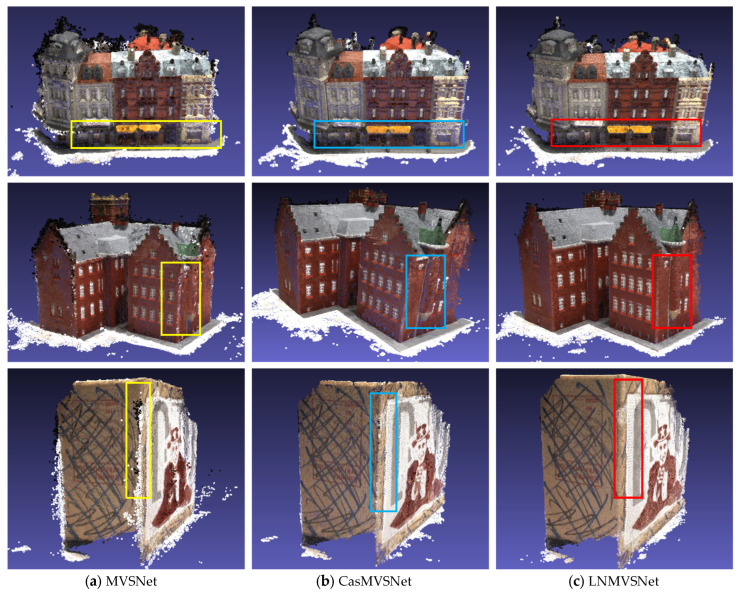
Visualization comparison of 3D reconstruction from different baselines on the DTU dataset.

The visual results (Figure 5) provide intuitive evidence for our quantitative analysis. Observing the 3D reconstruction effects of three distinct methods, LNMVSNet’s advantage in detail is more apparent. Whether it is the clarity of the edges of building windows or the authentic reproduction of surface textures, LNMVSNet exhibits higher quality and coherence. Particularly in the geometric details of buildings, LNMVSNet is capable of reconstructing smoother and more accurate surfaces, whereas the other methods experience blurriness or fragmentation in these areas. The ability of LNMVSNet to generate models with a low-noise profile is particularly evident in the visual outcomes, as it does not suffer from the extensive holes and outliers that are apparent in the outputs from networks like R-MVSNet and CasMVSNet, resulting in smoothly reconstructed object edges. It effectively minimizes the occurrence of outliers, ensuring that the reconstructed points accurately represent the true surface of the object without spurious data points that could potentially distort the model. Furthermore, the presence of fewer holes indicates a more continuous and cohesive data representation. This suggests that LNMVSNet’s sophisticated design is effective in achieving low-noise results in MVS 3D reconstruction. Its proficiency in handling noise and detail-rich scenes positions it at the forefront of the current 3D reconstruction networks. These outcomes strongly suggest that LNMVSNet is a network with significant advantages in accuracy, completeness, and visual quality, making it especially suited for applications that demand high-quality 3D models.

### 5.3. Tank and Temple Quantitative Results

**Figure 6:**
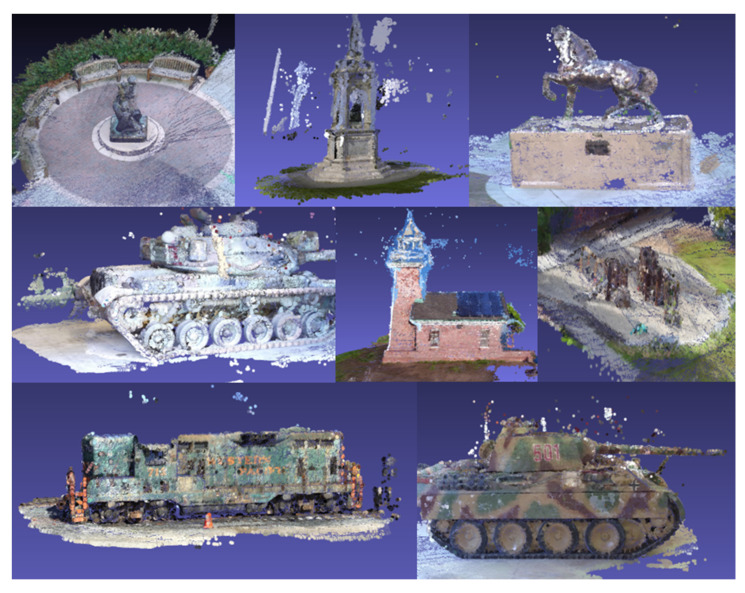
Visualization results of LNMVSNet on the Tank and Temple benchmark dataset.

To substantiate the network’s generalizability, we also conducted quantitative experiments on the Tank and Temple dataset, with the results presented in Table 2 and Figure 6. Table 2 presents a quantitative comparison of LNMVSNet against various state-of-the-art (SOTA) algorithms, employing a percentage metric, where higher values denote superior quality. It is observed that LNMVSNet achieves relatively high scores across multiple datasets, particularly notable on the “Family” and “Francis” datasets, with scores of 76.77% and 59.95%, respectively, significantly surpassing other methods. The mean score of LNMVSNet stands at 60.44%, indicating its ability to maintain consistently high performance across diverse scenarios.

Figure 6, on the other hand, showcases the complete reconstruction visual results of LNMVSNet on the Tank and Temple benchmark dataset. The visualization results from the Tank and Temple benchmark dataset reflect that the reconstructed 3D models are rich in detail, with clear surface textures. For instance, even the smaller components on the models of tanks and trains are precisely reconstructed, demonstrating LNMVSNet’s capacity to preserve high-quality reconstruction effects when processing low-noise data.

In summary, LNMVSNet’s performance in low-noise multi-view stereo (MVS) reconstruction is quite impressive. It not only provides a wealth of details and high-quality textures in the visual outcomes but also exhibits a reconstruction quality that surpasses other methodologies, especially in datasets of higher complexity.

## 6. Qualitative Analysis

**Figure 7:**
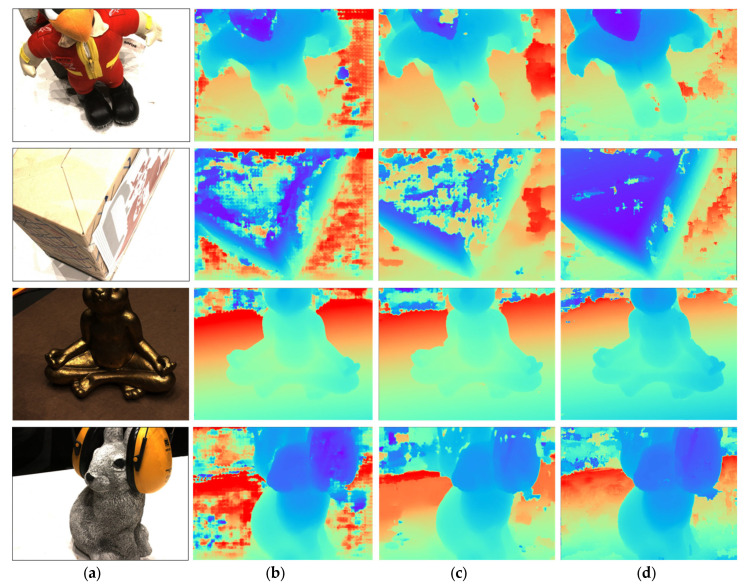
Visualization results of depth map generation from different baselines on the DTU dataset: (**a**) Reference Image; (**b**) MVSNet; (**c**) CasMVSNet; (**d**) LNMVSNet.

In this section, we incorporate qualitative experiments to explore the specific impact of noise as a factor in the network. Given that LNMVSNet is based on depth map reconstruction in multi-view stereo (MVS) methods, we print out the depth maps from LNMVSNet and display them in Figure 7 and calculate qualitative metric results in Table 3 for comparison with depth maps from classical depth reconstruction methods. It is evident from the images that LNMVSNet’s depth estimation on the DTU dataset avoids most of the voids, outliers, and edge inconsistencies. LNMVSNet ensures accuracy for the majority of pixels in depth estimation and maintains very clear edges. In the evaluated scenarios, LNMVSNet demonstrates the lowest average error and higher performance percentages across all listed error thresholds, particularly excelling with a performance of 91.8% under the error threshold of less than 8 mm^2^. CasMVSNet performs best at the error threshold of less than 2 mm^2^, achieving 82.6%. MVSNet shows the lowest performance across all metrics. This indicates that under these evaluation conditions, both LNMVSNet and CasMVSNet outperform MVSNet in terms of accuracy and efficiency.

To quantify the noise level in depth maps, the Blockiness factor [41] is primarily used to measure the image artifacts due to pixel and block distortions, especially in images post-downsampling. There are various methods for calculating the Blockiness factor, a common approach being the computation of differences in edges between adjacent blocks, namely, calculating both Horizontal and Vertical Blockiness. The steps are as follows: firstly, calculate the differences in edges between horizontally adjacent pixel blocks. Here, I(i,j) represents the pixel value at position (i,j) in the image, with M and N being the image’s height and width in pixels, respectively, and $N_{h}$ is the total number of horizontal edges.

(13) $$B_{h}=\frac{1}{N_{h}}\sum_{i=1}^{M}\sum_{j=1}^{N-1}\left|I\left(i,8j\right)-I\left(i,8j+1\right)\right|\;$$

where 8 serves as a stride or block size. In the computation of Blockiness, the analysis is conducted by traversing and calculating the differences between adjacent pixel blocks, with each block being approximately 8 pixels in size. The differences in edges between vertically adjacent pixel blocks are calculated similarly. The formula for this can be expressed as follows, where $N_{v}$ is the total number of vertical edges:

(14) $$B_{v}=\frac{1}{N_{v}}\sum_{i=1}^{M-1}\sum_{j=1}^{N}\left|I\left(8i,j\right)-I\left(8i+1,j\right)\right|\;\;$$

The overall Blockiness factor is a combination of the Horizontal and Vertical Blockiness, and it can be computed by taking the average of these two values. A lower value of the overall Blockiness factor indicates fewer discordant areas in the image, signifying better image quality. Conversely, a higher value implies the presence of noticeable noise structures or discontinuities in edges, which typically degrade visual quality. We ultimately normalized the Blockiness factor and presented the results in Table 4.

## 7. Conclusions and Future Work

The introduction of LNMVSNet marks a significant advancement in the field of multi-View stereo (MVS) 3D reconstruction, specifically in the effective removal of noise and qualitative analysis within the task of MVS stereo matching depth estimation. By employing strategies that enhance local feature attention and fuse features across different scales, LNMVSNet successfully overcomes the limitations encountered by traditional and deep learning-based methods in noise handling. The superior performance of LNMVSNet is not only evident in the improved accuracy and completeness of the reconstructed models but also in its ability to recover fine details and delineate clear feature boundaries, offering new possibilities for MVS applications across various industries. From precise industrial inspections to the creation of immersive virtual environments, LNMVSNet heralds the wide-ranging application prospects of this technology, paving new paths for future research and application. 

However, despite the exemplary performance demonstrated by LNMVSNet across multiple benchmark datasets, its generalization to real-world data of varying sources and quality remains a challenge. Future research may need to explore ways to further enhance the model’s adaptability to images under different environmental and lighting conditions, along with conducting more experiments on real-world datasets. Similarly, the performance limits of LNMVSNet under extreme noise conditions or highly complex scenarios have not been fully explored. An in-depth investigation into the model’s robustness under extreme conditions is a direction for future work.

## Figures and Tables

**Table 1:** Quantitative comparison results of point cloud 3D reconstruction on the DTU dataset.

Methods	Acc. (mm)	Comp. (mm)	Overall ^1^
Colmap [8]	0.400	0.664	0.532
Tola [10]	0.342	1.190	0.766
Furu [15]	0.612	0.939	0.775
Gipuma [19]	0.283	0.873	0.578
Colmap	0.400	0.644	0.532
MVSNet [22]	0.456	0.646	0.551
CasMVSNet [23]	0.325	0.385	0.355
EPPNet [24,25]	0.413	0.296	0.355
CVP-Net [25]	0.296	0.406	0.351
PatchNet [26]	0.427	0.277	0.352
R-MVSNet [27]	0.383	0.452	0.417
EP-Net [29]	0.299	0.323	0.311
LNMVSNet	0.305	0.311	0.308

^1^ Lower Acc., Comp., and Overall, by using the distance metric [mm], indicates better quality.

**Table 2:** Quantitative comparison results on the Tanks and Temples dataset using percentage metric.

Method	Family	Francis	Horse	Lighthouse	M60	Panther	Playground	Train	Mean ^1^
COLMAP [8]	50.41	22.25	26.63	56.43	44.83	46.97	48.53	42.04	42.14
ACMM [9]	69.24	51.45	46.97	63.20	55.07	57.64	60.08	54.48	57.27
PatchNet [26]	66.99	52.64	43.24	54.87	52.87	49.54	54.21	50.81	53.15
R-MVSNet [27]	73.01	54.46	43.42	43.88	46.80	46.69	50.87	54.25	50.55
CasMVSNet [23]	76.37	58.45	46.26	55.81	56.11	54.06	58.18	49.51	56.84
Vis-MVSNet [39]	77.40	60.23	47.07	63.44	62.21	57.28	60.54	52.07	60.03
MVSCRF [40]	59.83	30.60	29.93	51.15	50.61	51.45	52.60	39.68	45.73
LNMVSNet	76.77	59.95	47.92	64.17	58.39	58.06	60.27	57.96	60.44

^1^ Higher value indicates better quality.

**Table 3:** Qualitative comparison results on the DTU dataset depth map by using mean error.

Method	Resolution	Mean Error ^1^	<2 mm ^2^	<4 mm	<8 mm
MVSNet	1/4	11.63	63.1%	79.95%	87.86%
CasMVSNet	1	8.30	82.6%	86.70%	90.10%
LNMVSNet	1	6.82	77.67%	85.65%	91.8%

^1^ Lower mean error indicates a lower noise factor. ^2^ Higher percentage indicates better performance.

**Table 4:** Qualitative comparison results on the DTU dataset depth map by using the Blockiness factor.

Methods	Blockiness Factor ^1^
MVSNet	0.76
CasMVSNet	0.61
LNMVSNetap	0.34

^1^ A lower value indicates lower noise.

## Data Availability

No new data were published in this article.
